# Long-Term Characteristics of Human-Derived Biliary Organoids under a Single Continuous Culture Condition

**DOI:** 10.3390/cells11233797

**Published:** 2022-11-27

**Authors:** Ranan G. Aktas, Michael Karski, Biju Issac, Liang Sun, Shira Rockowitz, Piotr Sliz, Khashayar Vakili

**Affiliations:** 1 Department of Surgery, Boston Children’s Hospital, Boston, MA 02472, USA; 2 Cancer and Stem Cell Research Center, School of Medicine, Maltepe University, Istanbul 34857, Turkey; 3 Research Computing, Information Technology, Boston Children’s Hospital, Boston, MA 02472, USA; 4 The Manton Center for Orphan Disease Research, Boston Children’s Hospital, Boston, MA 02472, USA; 5 Division of Molecular Medicine, Boston Children’s Hospital, Boston, MA 02472, USA

**Keywords:** 3D, biliary system, extracellular matrix, microenvironment, organoid, primary cells, three dimensional

## Abstract

Organoids have been used to investigate the three-dimensional (3D) organization and function of their respective organs. These self-organizing 3D structures offer a distinct advantage over traditional two-dimensional (2D) culture techniques by creating a more physiologically relevant milieu to study complex biological systems. The goal of this study was to determine the feasibility of establishing organoids from various pediatric liver diseases and characterize the long-term evolution of cholangiocyte organoids (chol-orgs) under a single continuous culture condition. We established chol-orgs from 10 different liver conditions and characterized their multicellular organization into complex epithelial structures through budding, merging, and lumen formation. Immunofluorescent staining, electron microscopy, and single-nucleus RNA (snRNA-seq) sequencing confirmed the cholangiocytic nature of the chol-orgs. There were significant cell population differences in the transcript profiles of two-dimensional and organoid cultures based on snRNA-seq. Our study provides an approach for the generation and long-term maintenance of chol-orgs from various pediatric liver diseases under a single continuous culture condition.

## 1. Introduction

Organoids are rapidly becoming a powerful tool in various areas of biomedical research [1,2,3,4,5]. They can recapitulate some of the in vivo architecture, spatial organization, and genetic diversity of the cell populations found in the original organ with remarkable fidelity [1,6,7,8]. Furthermore, these three-dimensional (3D) structures can be grown in vitro to produce miniaturized versions of the tissues from which they were derived. Therefore, these self-organizing 3D structures offer a distinct advantage over traditional two-dimensional (2D) culture techniques by creating a more physiologically relevant milieu to study complex biological systems.

The liver is a complex vital organ that plays a significant role in metabolic, synthetic, and detoxification processes. The primary cell in the liver is the hepatocyte. However, the role of the cholangiocytes, sinusoidal endothelial cells, Kupffer cells, and stellate cells is paramount for the liver’s normal function. The study of hepatocytes and cholangiocytes has historically been limited due to their short-term survival in two-dimensional cultures [9,10]. Since the development of the intestinal organoid culture system by the Clevers lab [11,12], 3D cultures have been increasingly utilized to study liver biology and model liver disease [1,7,11,13]. A significant focus of the liver organoid field has been on differentiating progenitor or stem cells into hepatocyte organoids (hep-orgs) using specific conditions since the establishment of primary hepatocyte cultures has historically been challenging [7,14]. Both hep-org and chol-orgs have been generated from bipotential progenitor cells [15]. The main approach for establishment of hep-orgs has been to first establish progenitor organoids which can be differentiated into cholangiocytic organoids (chol-orgs) or hep-orgs [7,11].

Much of the previous work has focused on altering the conditions of progenitors or early organoids to induce differentiation into hepatocytes. However, in our work, we only focused on chol-orgs. The goal of this study was to determine the feasibility of establishing organoids from various pediatric liver conditions and characterize the long-term evolution of chol-orgs under a single continuous culture condition without passaging, which is in contrast with the methodology of the majority of well-known studies which passage the organoids periodically (every 5–14 days) [7,16,17].

## 2. Materials and Methods

### 2.1. Organoid Culture

Human liver samples were obtained from patients undergoing liver transplantation or liver resection at Boston Children’s Hospital under an approved Institutional Review Board protocol (IRB-P00004293). Organoids were generated from the livers of 14 patients. Fresh liver tissue from each patient was sliced into approximately 1 mm cubes and digested with Gibco Liver Digestion Medium (ThermoFischer Scientific, Waltham, MA, USA) on a rotator and subsequently filtered through 60 μm cell strainers. The retained material on the strainer was digested with Accutase (MilliporeSigma, Burlington, MA, USA) on a shaker and centrifuged at 700× *g* for 10 min. The pellet was resuspended in DMEM and centrifuged at 700× *g* for 5 min. The pellets were embedded in 50 μL Matrigel (Corning, Glendale, AZ, USA) drops in 24-well plates. The cells were maintained in advanced DMEM/F12 supplemented with Rspo1 (500 ng/mL; PeproTech, Cranbury, NJ, USA), Wnt3a (100 ng/mL; R&D Systems, Minneapolis, MN, USA), Noggin (100 ng/mL; Peprotech), HEPES (1 M; ThermoFisher Scientific), Antibiotic Antimycotic Solution (10 mL/L; Sigma-Aldrich, St. Loius, MO, USA), EGF (100 ug/mL Peprotech), Gastrin (10 μM; Sigma-Aldrich), HGF (0.3 ug/mL; Peprotech), A8301 (5 mM; Sigma), Rspo3 (100 ug/mL; Peprotech), and Y27632 (10 μM; Sigma-Aldrich). The media was renewed every 2–3 days. Two-dimensional (2D) cultures were formed spontaneously when the organoids resided along the Matrigel and culture dish interface. Once a colony of 2D cells was established, the Matrigel was removed, and the culture was maintained as a 2D culture using the media composition described above.

### 2.2. Immunohistochemistry

A whole-mount indirect immunolabeling methodology was used. Briefly, organoids were fixed by immersion in 4% paraformaldehyde and immunolabeled with the proper primary and secondary antibodies. Antibodies used in this study consisted of: AFP (Abcam, Waltham, MA, USA; cat. No. ab46799), Albumin (Fisher Scientific, cat. No. A80-229A), Beta-catenin (Cell signaling, Danvers, MA, USA; cat. No. 9562L), CFTR (Cell Signaling, cat. No. 78335S), CK19 (Abcam, cat. No. ab52625), EpCAM (Life Technologies, Carlsbad, CA, USA; cat. No. 14-9326-82), HNF4alpha (Abcam, cat. No. ab41898), OCT4 (Abcam, cat. No. ab18976), SOX9 (Abcam, cat. No. 76997), TOMM20 (Novus Biologicals, Centennial, CO, USA; cat. No. NBP2-67501), Vimentin (Cell Signaling, cat. No. 5741), and YAP (Cell Signaling, cat. No. 4912S). Secondary antibodies (Jackson ImmunoResearch, West Grove, PA, USA) included Alexa 488-anti Rabbit (cat. No. 711-545-152), Alexa 488-anti Mouse (cat. No. 715-116-150), Alexa 647-anti Rabbit (cat. No. 711-605-152), Alexa 594-anti Rabbit (711-585-152), Cy3 anti-goat (cat. No. 705-165-147), and Cy3 anti-rabbit (cat. No. 711-165-152). After washing with PBS, Vectashield mounting medium was used for DNA staining. The images of the organoids were obtained using confocal microscopy (Zeiss LSM 880; Dublin, CA, USA).

### 2.3. Microscopy

Organoid size, count, and morphology were assessed using brightfield and phase contrast imaging every other day. Organoids were fixed for at least 2 h at RT in the fixative (2.5% Glutaraldehyde 1.25% Paraformaldehyde and 0.03% picric acid in 0.1 M sodium cacodylate buffer (pH 7.4)). Consequently, they were washed in 0.1M cacodylate buffer, and postfixed with 1% Osmium tetroxide (OsO4)/1.5% potassium ferrocyanide (KfeCN6) for 1 h, washed 2× in water followed by Maleate buffer (MB) wash, and incubated in 1% uranyl acetate in MB for 1hr followed by two washes in water and subsequent dehydration in grades of alcohol (10 min each; 50%, 70%, 90%, 2 × 10 min 100%). The samples were then placed in propylene oxide for 1 h and infiltrated overnight in a 1:1 mixture of propylene oxide and Spurr’s low viscosity resin (Electron Microscopy Sciences, Hatfield, PA, USA). The following day the samples were embedded in Spurr’s resin and polymerized at 60 °C for 48 h in preparation for electron microscopy. Semi-thin sections were stained with Toluidine blue. After defining the best regions for ultrastructural examinations, ultrathin sections (about 80 nm) were cut on a Reichert Ultracut-S microtome, picked up onto copper grids stained with lead citrate, and examined in a JEOL 1200EX Transmission electron microscope images were recorded with an AMT 2k CCD camera (Advanced Microscopy Techniques, Woburn, MA, USA).

### 2.4. Single-Nucleus Transcriptome Analysis 

Organoids cultures were placed on ice to liquefy the Matrigel and then subjected to mechanical disruption via pipetting the media in the well. The organoids were further dissociated using a 25-gauge needle and resuspended in media before centrifugation at 300× *g* for 5 min. Following removal of the supernatant, the pellet was re-suspended in media and transferred immediately to Center for Cancer Genomics at Dana-Farber Cancer Institute (Boston, MA, USA) where single-nucleus RNA sequencing (snRNA-seq) was performed. Nuclei isolation was performed as previously described [18]. Low-retention microcentrifuge tubes (Fisher Scientific, Hampton, NH, USA) were used throughout the procedure to minimize nuclei loss. Briefly, organoids were homogenized in TST solution, filtered through a 30 mm MACS SmartStrainer (Miltenyi Biotec, Bergisch Gladbach, North Rhine-Westphalia, Germany), and pelleted by centrifugation for four minutes at 500× *g* at 4 °C. The nuclei pellet was resuspended in 200 uL of ST-SB buffer, and nuclei were counted by eye using INCYTO C-Chip Neubauer Improved Disposable Hemacytometers (VWR International Ltd., Radnor, PA, USA). Approximately 8,000 nuclei per sample were loaded per channel of the Chromium Next GEM Chip G for processing on the 10× Chromium Controller (10x Genomics, Pleasanton, CA, USA) followed by cDNA generation and library construction, as per manufacturer’s instructions (Chromium Next GEM Single Cell 3ʹ Reagent Kits v3.1 User Guide, Rev D). Library quality was assessed using a Bioanalyzer High Sensitivity DNA Analysis (Agilent Technologies, Lexington, MA, USA). Next, libraries were normalized and pooled in equal mass for sequencing on a NextSeq 150 cycle Mid-Output flow cell (Illumina, Inc., San Diego, CA, USA).

The raw single-nucleus sequencing data were preprocessed to generate the read count per gene per cell expression matrices using CellRanger (v6.1.1, 10x Genomics, San Francisco, CA, USA)). This includes BCL-to-FASTQ file conversion and data demultiplexing according to cell barcodes using the cellranger mkfastq function, genome alignment to human genome GRCH38, and read counting per gene per cell into cellxgene expression matrices using cellranger count function (include-introns). An R-based pipeline for the single-cell profiling was built based on the Seurat R toolkit (R v3.6.2, Seurat v3.1.2) [19,20]. The pipeline includes steps for cell filtering, clustering, annotation, differential gene expression, and visualization. Cells expressing 200 to 2500 genes that were detected in at least three cells and have less than 5% of mitochondrial genes detected in droplets were considered viable and singlets to be included for further analysis. After processing to extract cells of good quality, the two experimental groups were integrated for analysis by identifying common sources of variation using canonical correlation analysis [19,20]. Data dimension reduction using principal component analysis (PCA) (dimensions = 20) was performed on integrated data prior to clustering. Cell clustering was performed with a K-nearest neighbor (KNN) graph-based method using FindNeighbors function, followed by the original Louvain algorithm for modularity optimization (resolution = 0.5) using FindClusters function. After the cell clusters were determined, their marker genes were identified with the FindMarkers function.

Clusters obtained from snRNA-seq analysis were annotated using singleCellNet (SCN) v0.1.0 R package (v4.0.2) [21]. The publicly available GSE115469 dataset, which has data for ~20k gene features from 8444 liver cells obtained from five human subjects [22], was downloaded from NCBI GEO database for our analysis. This dataset has annotations for 20 different cell types. Gene expression count matrices from MacParland dataset was split into training and validation datasets as per developer defaults. SCN normalizes scales and then extracts the top classification genes and gene pairs for each cell type to train and build a multi-class top-pair Random Forest (TP-RF) classifier. SCN determines the top 50 most differentially expressed genes per cell type using a linear regression method, while the top 100 gene-pair per cell type are ranked using transformed pairwise expression correlation from those top DE genes [21]. SCN uses the top 50 most differentially expressed genes and the top 100 gene-pairs to build the classifier for training. Classifier performance was assessed using Cohen’s kappa (0.92) and mean AUPRC (0.98). Cohen’s kappa measures the agreement between the predicted and validation datasets while AUPRC measures the preciseness of the predictions. In general, the individual cells in data for which true identity cannot be determined by SCN were categorized as a ‘random’ cell type by the classifier.

## 3. Results

### 3.1. Chol-Orgs Were Established from Patients with a Variety of Liver Disorders

We attempted to establish liver-derived organoids from 14 patients using discarded liver tissue following liver transplantation. Patient diagnoses and organoid characteristics are listed in Table 1.

We were able to establish liver-derived organoids from 10 patients. Patient 8 had a diagnosis of hepatoblastoma, and we were able to establish organoids from the tumor sample but not from the adjacent normal liver. The rate of organoid growth varied between different patients; however, we were able to maintain organoid cultures for over six months using a single media formulation. We termed organoids established within 2 weeks of culture initiation as early-stage organoids and those beyond 60 days as late-stage organoids.

### 3.2. Microscopic Analysis of Chol-Orgs Revealed the Formation of Complex Epithelial Structures with Compartmentalization over Time

**Figure 1:**
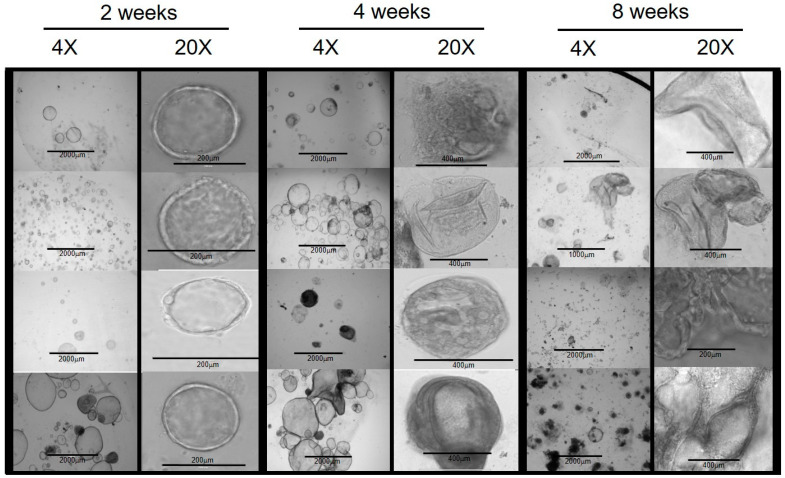
Chol-org morphology over 8 weeks from 4 different patient samples with underlying liver disease (from top to bottom: PFICIII, autoimmune hepatitis, alpha-1 antitrypsin deficiency, and ornithine transcarbamylase deficiency). At 2 weeks, chol-orgs demonstrate a simple spheroidal shape which develops into multi-layered and complex structure by 8 weeks.

Brightfield and phase-contrast microscopic examinations of developing organoids were performed at 1 to 3-day intervals. In the early period, within 2 weeks of establishing the initial culture, the organoids demonstrated a spheroid shape with varying sizes ranging from 60 μm to 600 μm (Figure 1).

**Figure 2:**
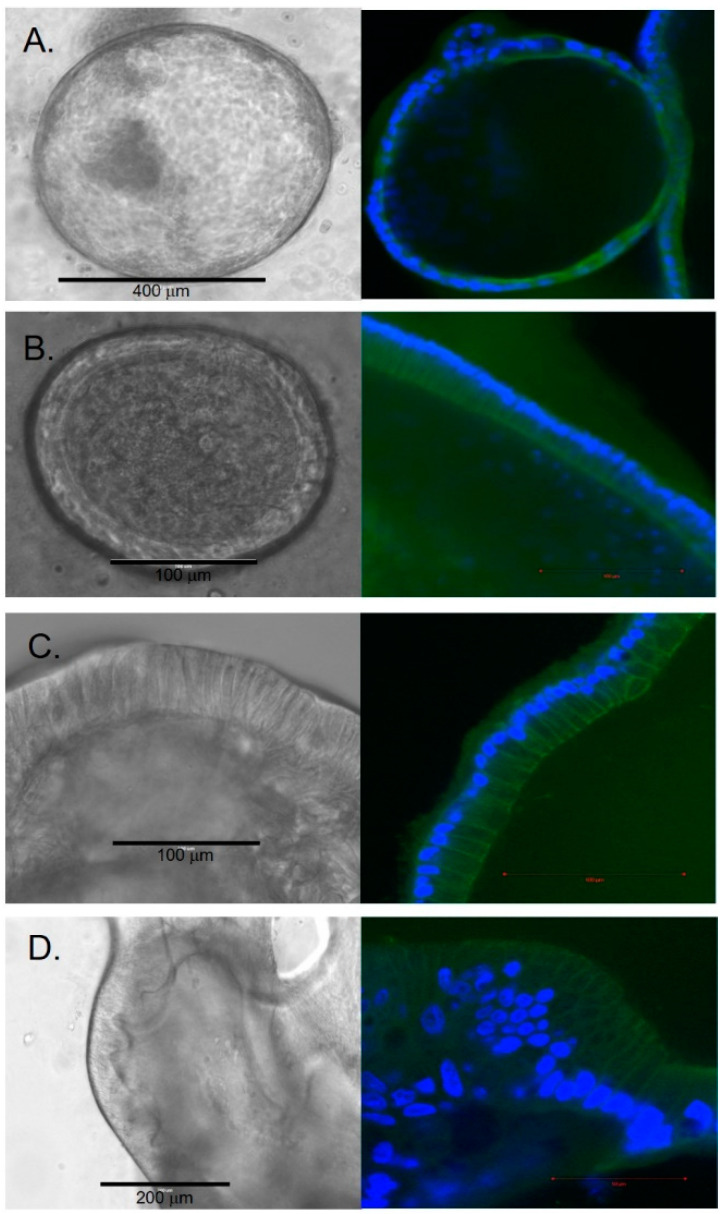
Cellular histology of chol-orgs with left column representing bright-field images and right column representing immunostaining with CK17 (green) and DAPI (blue) (**A**) Squamous epithelial morphology is present in early chol-orgs. Over time, cells develop a columnar epithelial morphology (**B**,**C**). In late organoids, multi-layered epithelial structures are observed (**D**).

The cellular composition of the spheroid structures had a histologic appearance of simple squamous epithelium. Over time, some organoids continued to enlarge, and the cells began to demonstrate a cuboidal and columnar histologic appearance (Figure 2). By 2 weeks, some organoids enlarged to a diameter of over 1000 μm. The growth rate within each well varied between individual organoids.

**Figure 3:**
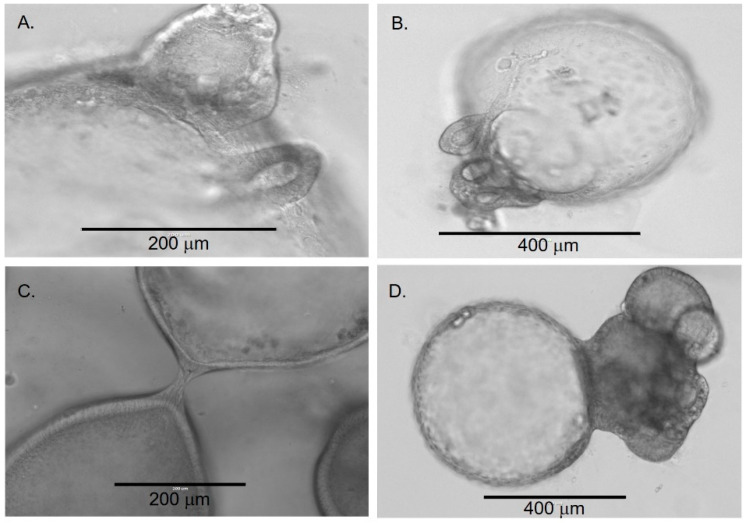
Transition of early chol-orgs into more complex forms. Budding (**A**,**B**) and merging (**C**,**D**) processes are observed as the chol-orgs transform into complex structures.

**Figure 4:**
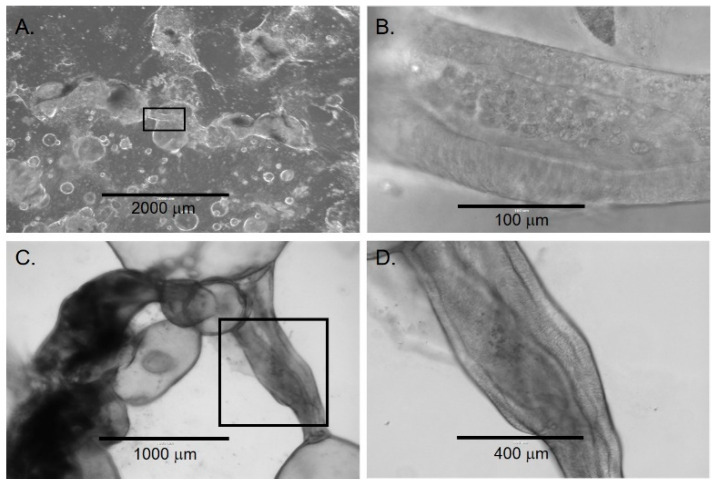
Lumen formation in late chol-orgs. (**A**) Low-magnification view demonstrates a culture with simple and complex chol-orgs and duct-like structure (black box) which is shown at higher magnification in panel (**B**). (**C**) Duct-like structure (black box) between two organoids with higher magnification in panel (**D**). Note the columnar epithelia along both lateral aspects of the lumen.

**Figure 5:**
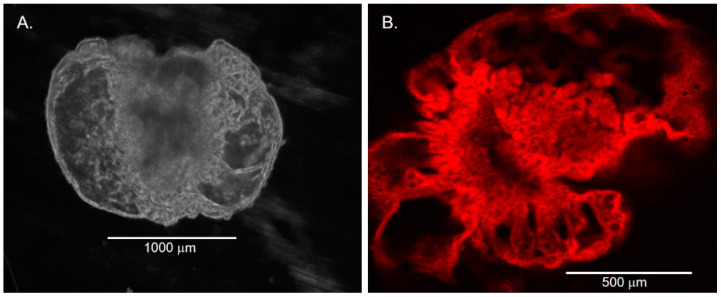
Compartment formation in late chol-orgs. (**A**) Phase contrast image demonstrate several compartments within a late chol-org. (**B**) IMMUNOFLUORESCENT imaging labeling CFTR.

Around four weeks, organoids began to demonstrate significant changes in their morphology. Some organoids developed areas of “budding,” which appeared as an eccentric mass of cells on the spheroidal organoids (Figure 3A,B). In addition, some organoids appeared to merge by establishing physical contact and further developing into a more complex structure (Figure 3C,D). The merging process appeared to start with the migration and organization of cells from one region on the organoid into a needle-like structure directed towards an adjacent organoid (Figure 3C). As the merging process continued, organoids developed a more dense and irregular appearance. The development of these denser organoids was associated with an increasing number of cuboidal and columnar epithelial cells. Furthermore, there was formation of duct-like structures between the organoids (Figure 3). Around eight weeks, the number of irregular and dense organoids continued to increase. The formation of duct-like structures (Figure 4) and multiple lumens or ‘compartmentalization’ within organoids was also observed (Figure 5). This was associated with an increased number of cellular layers, from one to two layers to 2 to 10 layers.

Over time, the majority of cultures contained structurally variable organoids ranging from simple, spherical organoids to organoids that were structurally more complex. To understand if the early-stage organoids transformed into late-stage organoids, we selected and tracked the early-stage organoids over time. We observed that some of the early organoids transformed into dense, irregular late-stage organoids (Figure 4 and Figure 5).

### 3.3. Organoid Immunostaining Revealed Specific Differences between Early-Stage and Late-Stage Organoids

**Figure 6:**
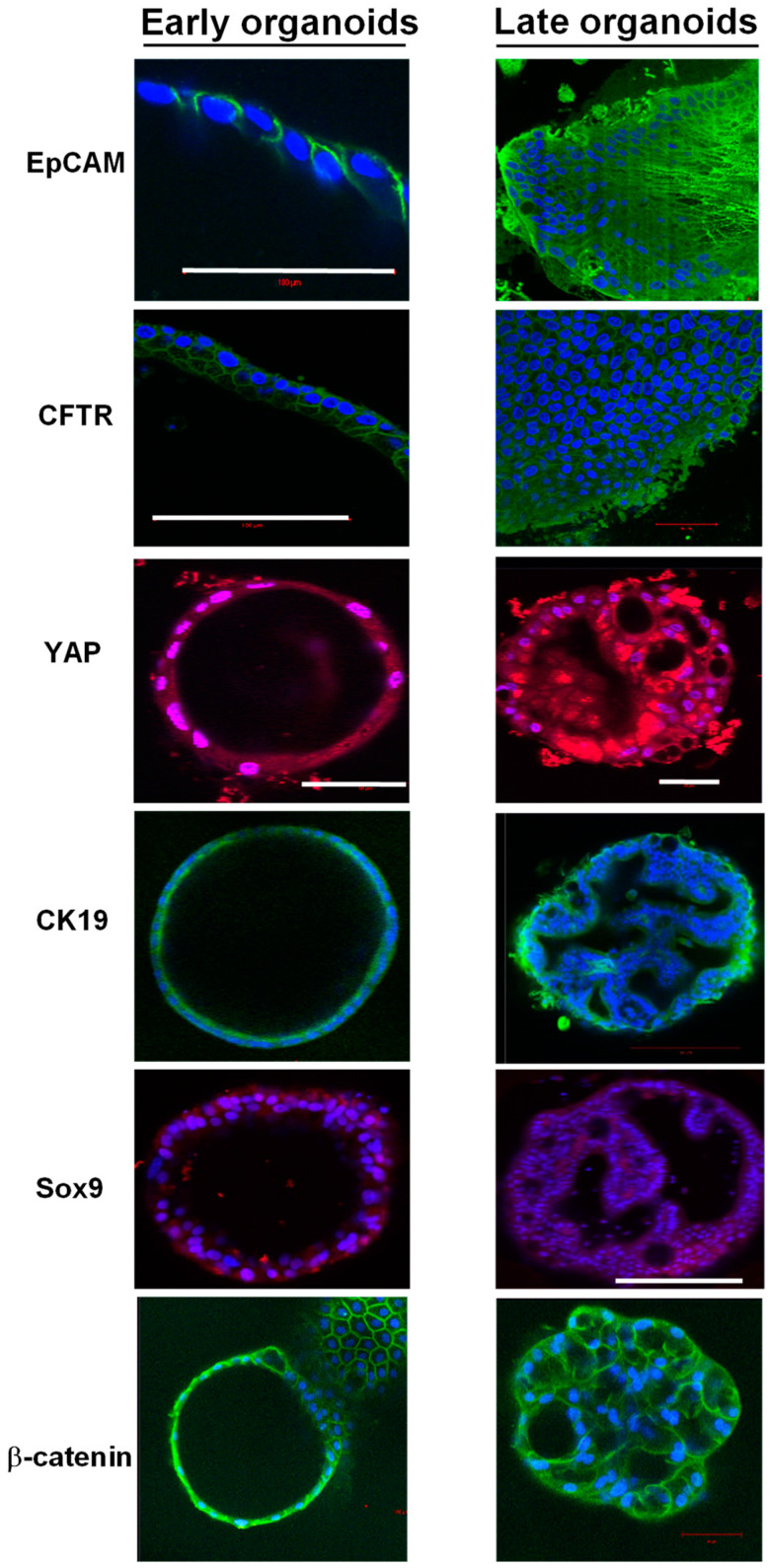
Immunofluorescence staining of early and late chol-orgs. EpCAM, CFTR, and CK19 membranous staining is present in both early and late organoids. YAP staining is predominantly nuclear in early organoids with increasing cytoplasmic staining in late organoids. Sox9 nuclear staining is patchy and weak in both early and late organoids. Membanous β-catenin staining is present in both early and late organoids. DAPI (blue) is used as a nuclear stain. Scale bars (white line): 100 μm (EpCAM and CFTR); 50 μm (YAP); and 200 μm (Sox9).

The expression and localization of 15 proteins of interest were assessed in early and late organoids using immunostaining (Figure 6). EpCAM was localized on the cell membranes of the cells both in early and late-stage organoids. CFTR localized at the apical surfaces of the epithelial cells in early organoids, while it was mainly peripheral in the late organoids. YAP localized to the nuclei of cells in early organoids and had a more cytoplasmic localization in late organoids. A small proportion of cells expressed vimentin in the early organoids; however, it became more prominent within the peripheral cells of late organoids. We used Tom20 staining to assess the localization of mitochondria in the cells, which revealed localization to the basal part of some cells, suggesting cellular polarization. Oct4 staining was sparse in both early and late organoids. The majority of cells in both early and late organoids demonstrated cytoplasmic CK19 staining. Nuclear localization of Sox9 was present in early organoids but was primarily absent in late organoids. Sub-membranous staining for beta-catenin was present in early organoids, with a subsequent decrease in staining in late organoids. The cells in either early or late-stage organoids did not express albumin, alfa-fetoprotein, or HNF4a.

### 3.4. Electron Microscopic Analysis of Organoids Revealed the Development of Fine Epithelial Cellular Ultrastructure

Toluidine-blue stained semi-thin sections revealed differential staining density between the cells’ luminal and basal surfaces suggesting the development of cellular polarity. In addition, necrotic changes were evident within the center of large late-stage organoids, reflecting a lack of nutrient penetration into the center of the complex structures.

**Figure 7:**
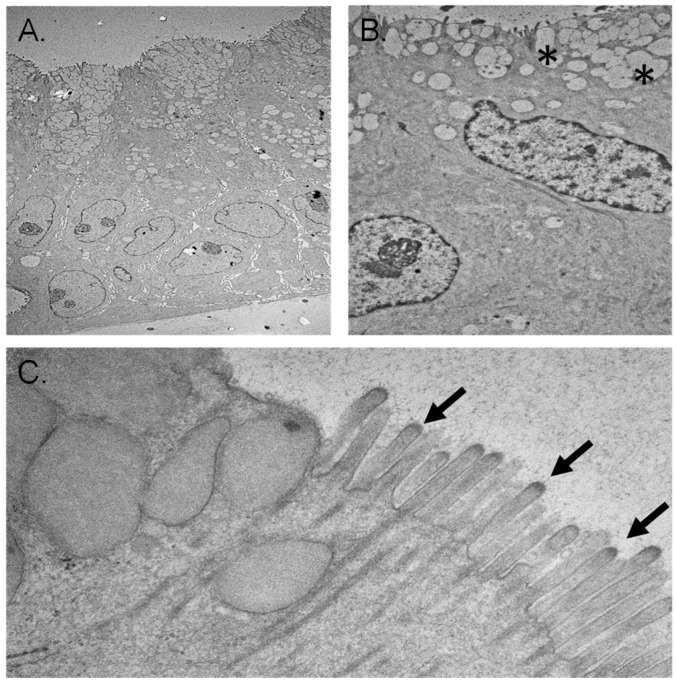
Electron microscopy of chol-orgs. (**A**) Luminal (top of panel) and basal surfaces (bottom of panel) are represented. The pattern of cell nuclei near the basal surface is consistent with a multilayer epithelial structure. (**B**) Higher magnification image demonstrates concentration of secretory vesicles (denoted by *) near the luminal border of the chol-org. (**C**) Cilia (arrows) are present along the luminal surface.

Ultrastructural examination of the early-stage organoids using electron microscopy revealed a single layer of polarized epithelial cells resting directly on the Matrigel scaffold. Squamous nuclei and scarce cytoplasmic organelles were evident. Secretory vesicles were present on the luminal surfaces, and some cells possessed microvilli. Junctional complexes between the adjacent cells were also clear (Figure 7).

Electron microscopic examination of late-stage organoids demonstrated the formation of different types of epithelial tissue ranging from simple squamous to stratified epithelium (Figure 7). The apical cytoplasm of some epithelial cells contained abundant secretory vesicles. These cells were interspersed between non-secretory epithelial cells. Microvilli and cilia formation on the luminal surface of some epithelial cells were evident (Figure 7). The basal cytoplasm of the cells contained abundant mitochondria, suggesting the development of cellular polarity. Intermediate filaments in the cytoplasm of some cells were also prominent. Sparsely, the formation of biliary canaliculi-like structures between cells was present.

### 3.5. Transcriptome Analysis Demonstrates Cholangiocytic Gene Signature of Chol-Orgs

**Figure 8:**
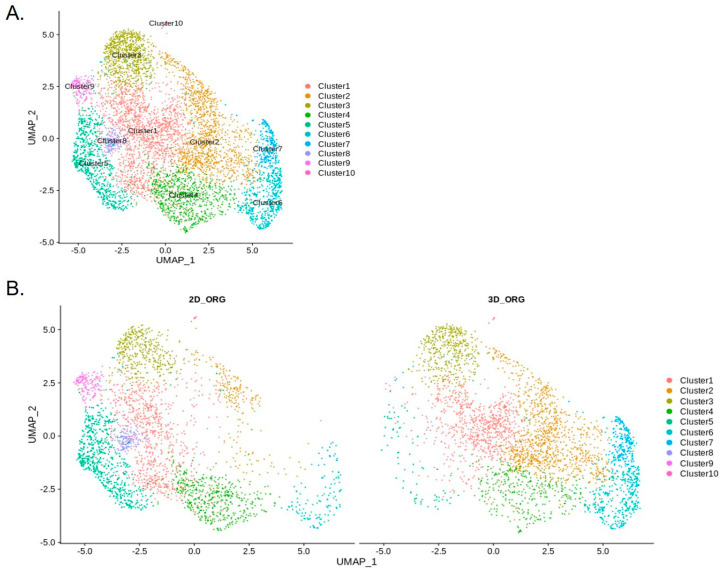
Unannotated UMAP clusters from snRNA-seq. (**A**) Integrated UMAP showing numbers of clusters detected in the snRNA-seq raw data, including both 2D and 3D cells. (**B**) Comparative UMAP plots showing differences in cell clustering patterns from snRNA-seq data based on 2D or 3D culture conditions. Cluster 2 cell population is significantly decreased in 2D chol-org culture whereas Clusters 5, 8, and 9 constitute a higher proportion of cells in 2D chol-org culture.

Single-nucleus RNA sequencing (snRNA-seq) of chol-org cultures identified 10 distinct clusters (Figure 8). Three-dimensional chol-orgs from Patient 11 were converted to 2D cultures in order to assess the transcriptomic alterations based on culture technique. Two-dimensional chol-org cultures were established by removing 3D organoids from Matrigel and transferring them to a standard two-dimensional culture system.

**Figure 9:**
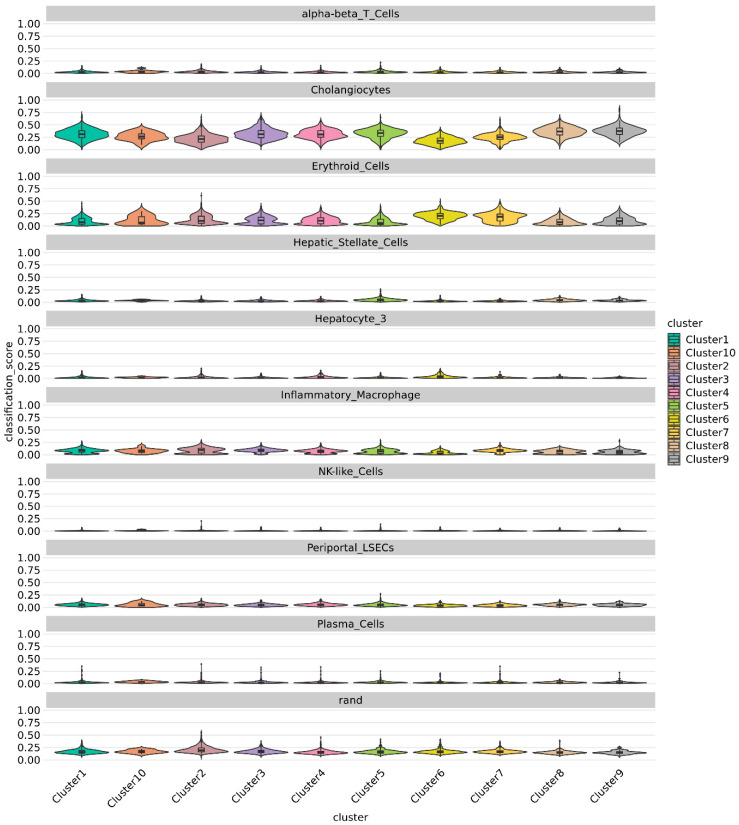
Violin plot showing prediction from singleCellNet for each cluster based on top 50 discriminant genes and top 100 gene pairs derived from cell types from MacParland [22] dataset classifier model. All Clusters demonstrate a strong cholangiocytic signature.

Using published single-cell transcriptome of human liver [22], we classified the cells in integrated 3D and 2D chol-org cultures via SingleCellNet (SCN) [21]. The SCN classifier trained using the top 50 most differentially expressed (DE) genes per cell type and top 100 gene pairs (see methods) from the McParland study [22], demonstrated a significant cholangiocytic profile in line with the reported results from McParland for all clusters (Figure 9). All clusters identified exhibited gene expression consistent with cholangiocytes, further suggesting that all of the cells differentiated appropriately into cholangiocytes.

**Figure 10:**
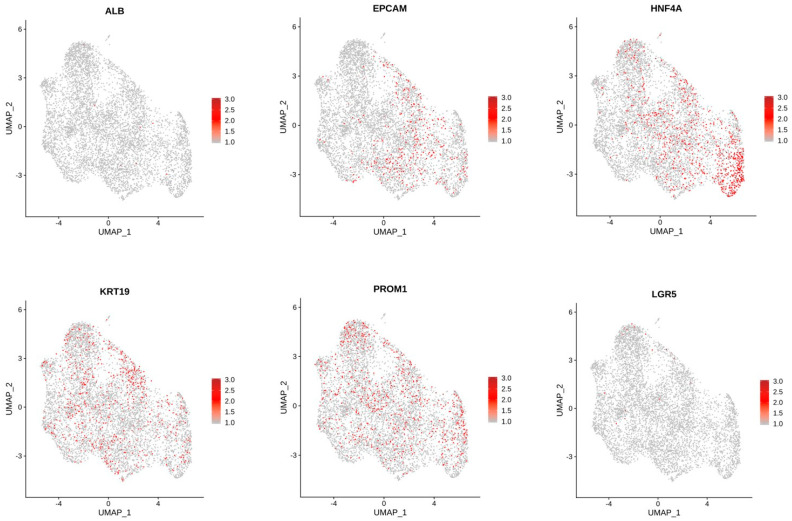
Feature Plots showing expression of genes of interest across cells in all clusters.

Comparison of cholangiocytes in the 3D and 2D cell clusters suggests differences in cell types between these two groups (Figure 8). The largest changes (Table 2) were in Cluster 2 (2D 7%; 3D 31%), Cluster 5 (2D 20%; 3D 2%), and Cluster 7 (2D 0.8%; 3D 8%), suggesting that there are significant differences in the transcriptional profiles of 2D and 3D cell differentiation. The top 10 genes in regard to statistically significant (*p* < 0.0005) expression differential in Cluster 2 between 3D and 2D conditions were (3D%, 2D% of cells): *NDRG1* (78%, 17%); *FAM13A* (77%, 19%); *PCAT* (13%, 67%); *FRY* (57%, 5%); *MUC13* (64%, 15%); *SLC6A8* (52%, 4%); *DUOX* (65%, 17%); *HIF1A* (65%, 17%) and *DPYD* (54%, 9%). The same analysis for Cluster 5 included the following genes (3D%; 2D%): *ANKRD1* (6%, 81%); *DCDC2* (24%, 90%); *FAM13A* (74%, 12%); *KRT7* (17%, 78%); *CDH20* (3%, 62%); *IL18* (15%, 72%); *UBASH3B* (22%, 79%); *FAM155A* (13%, 67%); and *NDRG1* (57%, 3%). Cluster 7 statistically significant (*p* < 0.05) differential gene expression between the two conditions revealed the following genes (3D%, 2D%): *KIAA1217* (13%, 69%); *IQGAP2* (39%, 89%); and *GOLM1* (39% 88%. Feature plots demonstrate KRT19 and PROM1 expression in all clusters (Figure 10).

## 4. Discussion

The ability to establish organoids from human liver progenitor cells has ushered in a new era in fundamental and translational research. Undoubtedly, human organoid systems will continue to improve and provide opportunities to study complex developmental mechanisms [1,2,13,16]. Here, we were able to generate and expand chol-orgs from pediatric liver tissue from a variety of liver diseases under a single continuous culture condition without passaging the cells. Our study is unique since the majority of previous long-term liver and biliary organoid studies were based on experiments performed after passaging and mechanical disruption of organoids at pre-determined intervals [7,11,16,17,23]. We maintained a single culture from each patient for a 2–6-month period. This approach allowed us to observe the changes in the organoids in a single and defined medium. To our knowledge, the current study represents the largest and longest observation of dynamic changes related to the phenotypic, ultrastructural, and transcriptomic features of chol-orgs derived from pediatric livers. The samples were derived from cirrhotic (e.g., biliary atresia, PFIC II, autoimmune hepatitis) and non-cirrhotic (e.g., Maple syrup urine disease, ornithine transcarbamylase deficiency) livers. Our sample size per disease type was not adequate to draw a statistically significant conclusion in regard to the inherent ability to establish organoids based on disease type. However, our results do suggest that chol-orgs can be established from a variety of liver pathologies. We maintained the cultures under a continuous WNT activating condition, and noted the three primary mechanisms of multicellular self-organization that are crucial for the emergence of latent intrinsic order: self-assembly, self-patterning, and self-driven morphogenesis [24].

Over several months, the early simple spheroid organoid underwent self-organization and transformation into multilayer organoids with several types of epithelial cells. We hypothesize that persistent activation of WNT signaling in chol-orgs result in stem-cell and progenitor-cell expansion, cell survival, the maintenance of epithelial architecture, and the promotion of intercellular interaction. Early chol-orgs demonstrated a spherical shape, which began to demonstrate budding and merging starting around 2 weeks following the initiation of the cultures. The organoids then progressed to form duct-like structures and compartments over subsequent weeks. The formation of different types of epithelial cells was evident with progression from squamous type epithelial cells to multilayered epithelium with columnar and cuboidal cells. In the more complex late chol-orgs secretory features were apparent with increasing cytoplasmic vesicles. In addition, cilia and microvilli formation on the apical surfaces of the cells suggest the development of cellular polarity and the construction of a barrier function. Our study is in line with the observation that immortalized cholangiocytes can build bile duct-like structures without extensive modification of media as reported by Lewis et al. [23] However, the duration of our cultures is longer than reported by that group.

Our study provides morphological data that demonstrate how a simple squamous epithelium turns into 10–12 multilayered epithelial tissue without mesenchymal tissue support and vascular network. We used Matrigel as a basement membrane model, given its property in promoting cell adhesion and differentiation. Laminin in the Matrigel acts as a dominant cue -over other molecules such as collagen type I and IV- to organize epithelial polarity and functions in epithelial morphogenesis. The ability for epithelial structures to form without mesenchymal niches had been previously described [12]. Interestingly, after separation and cultivation of early-stage organoids, we observed that some of the organoids would not expand and stayed at the same stage for the duration of the long-term culture. The reason for this finding and whether it is related to the microenvironment of the organoid is unclear.

Characterization of the chol-orgs using immunostaining demonstrated expression of biliary cell markers such as EpCAM and KRTImmunostaining for SOX9 revealed faint patchy nuclear staining and mainly cytoplasmic staining in early and late organoids. YAP was localized in the nuclei of the cells in early-stage organoids, and shifted to the cytoplasm of the cells in late-stage organoids. YAP is an effector of the Hippo pathway and plays a role in cell differentiation, proliferation, and migration [25,26]. We previously reported on the pattern of YAP expression during liver regeneration [27] and in hepatocellular carcinoma (HCC) [28] and noted that during regeneration, hepatocytes predominantly express nuclear YAP whereas in HCC, we observed significant cytoplasmic YAP in addition to nuclear YAP. The YAP staining we observed in late chol-orgs was very similar to that in previously reported HCC cells [28], though the exact function of this pattern of YAP localization is not known. YAP distribution between the nucleus and cytoplasm is known to be dynamic [27] and the chol-org model may provide a platform for elucidating YAP function in differentiating biliary cells. EpCAM, an epithelial adhesion molecule, was evident on the membranes of the cells both in early- and late-stage organoids. It is a marker for stem cells and tumor-initiating cells [29,30]. A novel connection between CFTR and epithelial morphogenesis and differentiation has been reported [31]. Our findings related to these markers support the organoids belonging to the cholangiocytic lineage. This is further supported by the single-cell nuclear RNA sequencing data which identified the cell types as cholangiocytes when compared to a previously published dataset characterizing the various cell populations in the liver [22]. Even though both the 2D and 3D chol-orgs demonstrated predominant cholangiocytic signatures, there were some notable gene expression differences between the two groups when the snRNA-seq clusters were analyzed in depth. Of note, *MUC13* was expressed in a significantly higher percentage of 3D chol-orgs compared to the 2D condition. *MUC13* is normally secreted or expressed on the apical surfaces of various epithelial cells for lubrication and protection of mucosal surfaces [32]. The higher expression of MUC13 in 3D chol-orgs is in line with lumen formation and development of apical and basal surfaces as observed on microscopic examination. Interestingly, *MUC13* expression has been associated with several types of cancers including cholangiocarcinoma [33], pancreatic [34], hepatocellular [35,36] and colorectal [36]. Other genes of interest with significantly higher expression in 3D chol-orgs compared to 2D cells were *NDRG1*, *FAM13A, FRY*, *HIF1A* and *DPYD*. *NDRG1* is a cytoplasmic and membrane-associated protein which may function in a tissue-specific manner and has been labeled as a metastasis-suppressor [37]. The membrane-associated NDRG1 has been found near adherens junctions, thereby suggesting a role in cell adhesion and adherens junction complex formation [38]. Interestingly, *NDRG1* has three hypoxia inducible factor 1 binding sites with one situated in its promoter, suggesting that *NDRG1* may be regulated by *HIF1A* [39,40], which is also significantly upregulated in the 3D chol-orgs. The upregulation of *HIF1A* and *NDRG1* in 3D chol-orgs raises the possibility that formation of the 3D epithelial structures under the described conditions may encounter relative hypoxia. Alterations in culture conditions to improve oxygen delivery to multicellular and complex structures may have a positive impact on 3D organoid culture methodology.

Our study presents the methodology and characterization of long-term biliary organoids from a variety of pathologic liver tissues in 3D culture without passaging the cells. This approach provides a model for studying complex epithelial tissue development under a single condition and over a longer period than previously reported.

## Figures and Tables

**Table 1:** Patient characteristics and organoid establishment.

Patient	Diagnosis	Age (Years)	Organoid Establishment
1	Biliary atresia	1	No
2	Biliary atresia	0.9	No
3	Maple syrup urine disease	11	No
4	Progressive familial intrahepatic cholestasis III	9	Yes
6	Autoimmune hepatitis	13	Yes
7	Alpha-1 antitrypsin deficiency	1	Yes
8a	Normal liver tissue (hepatoblastoma)	11	No
8b	Hepatoblastoma	11	Yes
9	Biliary atresia	1.5	Yes
10	Progressive familial intrahepatic cholestasis III	4	Yes
11	Ornithine transcarbamylase deficiency	2	Yes
12	Normal liver tissue (hepatoblastoma)	3.5	Yes
13	Fibrolamellar carcinoma	14	No
14	Congenital glycosylation type 1d, mannosyltransferase 6 deficiency	2.5	Yes
15	Progressive familial intrahepatic cholestasis II	6	Yes
16	Fibrolamellar carcinoma	15	Yes

**Table 2:** The number and percentage of cells in each cluster and condition.

Cluster IDs	2D Chol-Org	3D Chol-Org
1	785 (26.78%)	778 (24.03%)
2	204 (6.96%)	1018 (31.45%)
3	412 (14.06%)	426 (13.16%)
4	475 (16.21%)	348 (10.75%)
5	598 (20.4%)	76 (2.35%)
6	101 (3.45%)	320 (9.89%)
7	23 (0.78%)	245 (7.57%)
8	159 (5.42%)	15 (0.46%)
9	165 (5.63%)	6 (0.19%)
10	9 (0.31%)	5 (0.15%)

## Data Availability

The data presented in this study are available on request from the corresponding author.
